# Modulated phases of graphene quantum Hall polariton fluids

**DOI:** 10.1038/ncomms13355

**Published:** 2016-11-14

**Authors:** Francesco M. D. Pellegrino, Vittorio Giovannetti, Allan H. MacDonald, Marco Polini

**Affiliations:** 1 NEST, Scuola Normale Superiore and Istituto Nanoscienze-CNR, Piazza dei Cavalieri 7, Pisa I-56126, Italy; 2 Department of Physics, University of Texas at Austin, Austin, Texas 78712-1081, USA; 3 Istituto Italiano di Tecnologia, Graphene Labs, Via Morego 30, Genova I-16163, Italy

## Abstract

There is a growing experimental interest in coupling cavity photons to the cyclotron resonance excitations of electron liquids in high-mobility semiconductor quantum wells or graphene sheets. These media offer unique platforms to carry out fundamental studies of exciton-polariton condensation and cavity quantum electrodynamics in a regime, in which electron–electron interactions are expected to play a pivotal role. Here, focusing on graphene, we present a theoretical study of the impact of electron–electron interactions on a quantum Hall polariton fluid, that is a fluid of magneto-excitons resonantly coupled to cavity photons. We show that electron–electron interactions are responsible for an instability of graphene integer quantum Hall polariton fluids towards a modulated phase. We demonstrate that this phase can be detected by measuring the collective excitation spectra, which is often at a characteristic wave vector of the order of the inverse magnetic length.

## Abstract (web summary)

High-mobility graphene can play host to exciton polaritons—hybrid matter–light particles, which can form into a state known as a quantum Hall polariton fluid. Here, the authors show that electron–electron interactions can act to destabilize this state and lead to the formation of a modulated phase.

Fluids of exciton polaritons, composite particles resulting from coupling between electron–hole pairs (excitons) in semiconductors and cavity photons, have been intensively investigated over the past decade^1^,^2^,^3^. Because of the light mass of these quasi-particles, exciton-polariton fluids display macroscopic quantum effects at standard cryogenic temperatures^1^,^2^,^3^, in stark contrast to ultracold atomic gases. Starting from the discovery of Bose–Einstein condensation of exciton polaritons in 2006 (ref. 4), these fluids have been the subject of a large number of interesting experimental studies exploring, among other phenomena, superfluidity^5^,^6^, hydrodynamic effects^7^, Dirac cones in honeycomb lattices^8^ and logic circuits with minimal dissipation^9^.

The isolation of graphene^10^—a two-dimensional (2D) honeycomb crystal of carbon atoms—and other 2D atomic crystals^11^ including transition metal dichalcogenides^12^,^13^ and black phosphorus^14^, provides us with an enormously rich and tunable platform to study light–matter interactions and excitonic effects in 2D semimetals and semiconductors. Light–matter interactions in graphene in particular have been extensively explored over the past decade with both fundamental and applied motivations^14^,^15^,^16^,^17^,^18^. Recent experimental advances have made it possible to monolithically integrate graphene with optical microcavities^19^,^20^, paving the way for fundamental studies of cavity quantum electrodynamics at the nanometre scale with graphene as the active medium. Progress has also been made using an alternate approach applied previously to conventional parabolic-band 2D electron liquids in semiconductor quantum wells^21^ by coupling graphene excitations to the photonic modes of a terahertz (THz) metamaterial formed by an array of split-ring resonators^22^.

When an external magnetic field is applied to a 2D electron liquid in a GaAs quantum well^23^ or a graphene sheet^24^,^25^, and electron–electron (e–e) interactions are ignored, transitions between states in full and empty Landau levels (LLs) are dispersionless, mimicking the case of atomic transitions in a gas. The cyclotron resonance of a 2D quantum Hall fluid can be tuned to resonance with the photonic modes of a cavity or a THz metamaterial^21^, thereby establishing the requirements for ‘cavity quantum Hall electrodynamics' (cQHED). Cavity photons have already been used to carry out spectroscopic investigations of fractional quantum Hall fluids^26^. cQHED phenomena present several important twists on ideas from ordinary atom-based cavity quantum electrodynamics because in this case, interactions between medium excitations are strong and long ranged. Furthermore the active medium can be engineered in interesting ways, for example by using, instead of a single 2D crystal, van der Waals heterostructures^27^,^28^,^29^ or vertical heterostructures, which include both graphene sheets and ordinary semiconductor quantum wells^30^,^31^.

In this article, we show that e–e interactions play a major qualitative role in graphene-based cQHED. Before describing the technical details of our calculations, let us briefly summarize the logic of our approach. The complex many-particle system of electrons in a magnetic field, interacting between themselves and with cavity photons, is treated within two main approximations. We use a quasi-equilibrium approach based on a microscopic grand-canonical Hamiltonian and treat interactions at the mean-field level. We critically comment on these approximations after ‘Results' section. Our approach is similar to that used in refs 32, 33, 34, except that simplifications associated with LL quantization allow more steps in the calculation to be performed analytically.

The problem of finding the most energetically favourable state of a graphene integer quantum Hall polariton fluid (QHPF) is approached in a variational manner, by exploiting a factorized many-particle wave function. The latter is written as a direct product of a photon coherent state and a Bardeen–Cooper–Schrieffer state of electron–hole pairs belonging to two adjacent LLs. We find that e–e interactions are responsible for an instability of the uniform exciton-polariton condensate state towards a weakly modulated condensed state, which can be probed experimentally by using light scattering. We therefore calculate the collective excitation spectrum of the graphene QHPF by employing the time-dependent Hartree–Fock approximation. We demonstrate that the tendency to modulation driven by e–e interactions reflects into the softening of a collective mode branch at a characteristic wave vector of the order of the inverse magnetic length.

## Results

### Effective model

We consider a graphene sheet in the presence of a strong perpendicular magnetic field 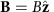 (refs 35, 36). We work in the Landau gauge with vector potential 
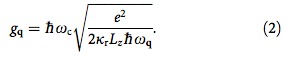
. The magnetic field quantizes the massless Dirac fermion (MDF) linear dispersion into a stack of LLs, 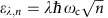, which are labelled by a band index *λ*=±, which distinguishes conduction and valence band states and an integer *n*=0,1,2,…. Here 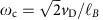 is the MDF cyclotron frequency^35^,^36^, 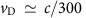 the Dirac band velocity (*c* being the speed of light in vacuum) and 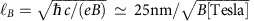 is the magnetic length. The spectrum is particle–hole symmetric, that is, *ɛ*_−,*n*_=−*ɛ*_+,*n*_ for each *n*. Each LL has macroscopic degeneracy 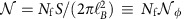, where *N*_f_=4 is the spin-valley degeneracy and *S*=*L*^2^ is the sample area.

In this article, we address the case of integer filling factors, which we expect to be most accessible experimentally. Because of particle–hole symmetry, we can assume without loss of generality that the chemical potential lies in the conduction band between the *n*=*M* and *n*=*M*+1 LLs. When the energy *ℏω* of cavity photons is nearly equal to the cyclotron transition energy 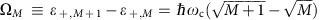, the full fermionic Hilbert space can be effectively reduced to the conduction-band doublet *M*,*M*+1.

We introduce the following effective grand-canonical Hamiltonian:


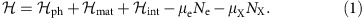


The first term, _ph_, is the photon Hamiltonian, 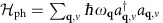, where 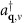 (*a*_*q*,*ν*_) creates (annihilates) a cavity photon with wave vector *q*, circular polarization *ν*=L,R and frequency 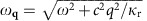, *κ*_r_ being the cavity dielectric constant and *c* the speed of light in vacuum.

The second term in equation (1), 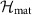, is the matter Hamiltonian, which describes the 2D MDF quantum Hall fluid, and contains a term due to e–e interactions. This Hamiltonian is carefully derived in the Supplementary Note 1. In brief, one starts from the full microscopic Hamiltonian of a 2D MDF quantum Hall fluid^36^, written in terms of electronic field operators *c*_*λ*,*n*,*k*,*ξ*_. Here, *λ*=± is a conduction/valence band index, *n* is a LL index, 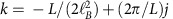 with 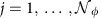 is the eigenvalue of the *x*-direction magnetic translation operator, and *ξ* is a fourfold index, which refers to valley (*K*,*K*^'^) and spin (↑,↓) indices. All the terms that involve field operators *c*_λ,*n*,*k*,*ξ*_, 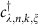 acting only on the conduction-band doublet *M*,*M*+1 are then treated in an exact fashion, while all other terms are treated at leading order in the e–e interaction strength^37^.

The third term, 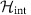, describes interactions between electrons and cavity photons, which we treat in the rotating wave approximation. This means that in deriving 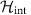 we retain only terms that conserve the sum of the number of photons and the number of matter excitations. Details can be found in the Supplementary Note 2. It is parameterized by the following light–matter coupling parameter


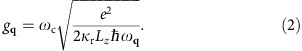


In equation (2) 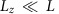 is the length of the cavity in the 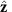 direction (*V*=*L*_*z*_*L*^2^ is the volume of the cavity). In what follows, we consider a half-wavelength cavity setting 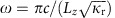. Consequently^25^, 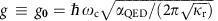, where 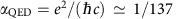 is the quantum electrodynamics fine-structure constant.

Finally, in equation (1) we have introduced two Lagrange multipliers, *μ*_e_ and *μ*_X_, to enforce conservation of the average number of electrons and excitations^38^. *N*_e_ is the electron number operator in the *M*,*M*+1 reduced Hilbert space, while *N*_X_=*N*_ph_+*N*_ex_ is the operator for the number of matter excitations (excitons). The value of the chemical potential *μ*_e_ should be fixed to enforce 〈*ψ*|*N*_e_|*ψ*〉=*N*. At zero temperature, this condition is simply enforced in the variational wave function defined below.

### Variational wave function and spin-chain mapping

To find the ground state of the Hamiltonian (1), we employ a variational approach in which the many-particle wave function |*ψ*〉 is written as^33^,^39^ a direct product of a photon coherent state and a Bardeen–Cooper–Schrieffer state of electron–hole pairs belonging to the *M*,*M* + 1 conduction-band doublet:


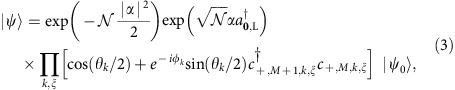


where |*ψ*_0_〉 is the state with no photons and with the *M*-th LL fully occupied. In writing equation (3), we have allowed for phase-coherent superposition of electron–hole pairs with *k*-dependent phases *φ*_*k*_ and excitation amplitudes sin(*θ*_*k*_/2), to allow for the emergence of modulated QHPF phases driven by e–e interactions. Equation (3) can be written in terms of polariton operators, as shown in Supplementary Note 3. The variational parameters {*φ*_*k*_}, {*θ*_*k*_}, and *α* can be found by minimizing the ground-state energy 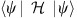. We introduce the following regularized energy (per electron)^40^:


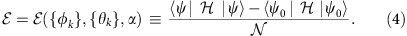


**Figure 1:**
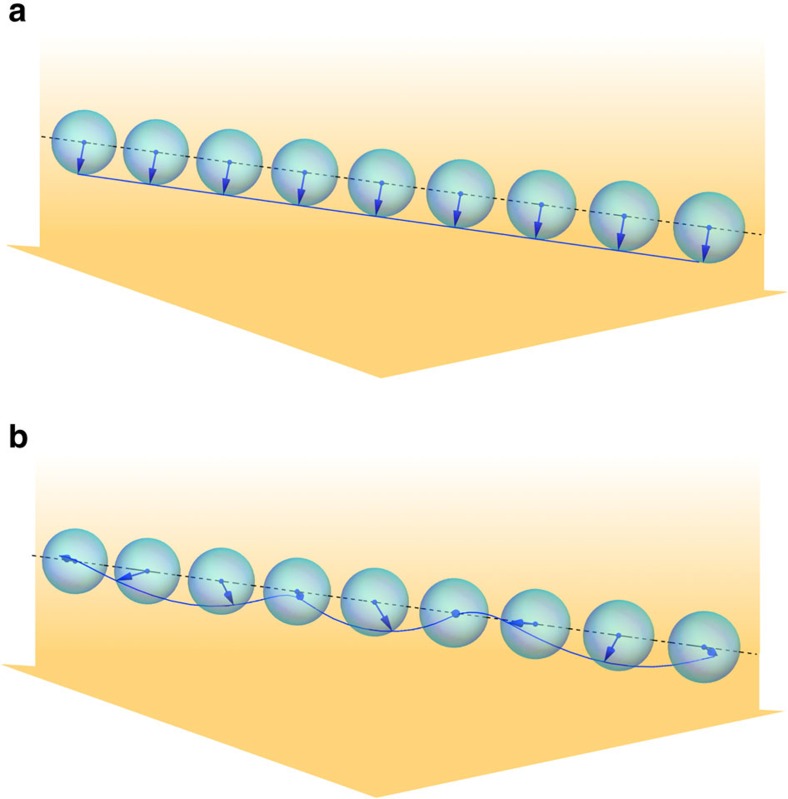
Phases of a graphene integer QHPF. Pictorial representation of the two phases supported by a graphene quantum Hall fluid interacting with a uniform electromagnetic field (in yellow). (**a**) When e–e interactions are weak, the ground state of the system |*ψ*〉 is a spatially uniform polariton condensate. In pseudospin magnetic language, this state is a collinear ferromagnet with all pseudospins, defined in equation (5), denoted by blue arrows in the Bloch sphere, pointing along a common direction. (**b**) When e–e interactions are sufficiently strong, the ground state of the system |*ψ*〉 spontaneously break translational invariance. In pseudospin magnetic language, this state is a spiral pseudospin state. The spiral is driven by antisymmetric interactions between and pseudospin components as explained in the main text.

The variational wave function (3) and the functional 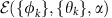 can be conveniently expressed in terms of the *k*-dependent Bloch pseudospin orientations:


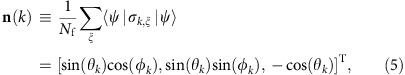


where 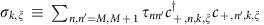 and *τ*=(*τ*_1_,*τ*_2_,*τ*_3_) is a three-dimensional vector of Pauli matrices acting on the *M*,*M* + 1 doublet. The variational wave function then becomes


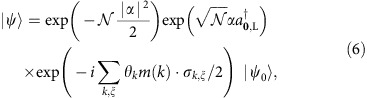


where **m**(*k*)=(sin(*φ*_*k*_), −cos(*φ*_*k*_),0)^T^ is a unit vector orthogonal to **n**(*k*) and |*ψ*_0_〉 contains all pseudospins oriented along the 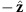 direction. Since exp[−*iθ*_*k*_**m**(*k*)·*σ*_*k*,*ξ*_/2] acts as a rotation by an angle *θ*_*k*_ around **m**(*k*), we can interpret the matter part of |*ψ*〉 as a state, in which every pseudospin labelled by (*k*,*ξ*) is rotated accordingly. The unit vector **n**(*k*) in equation (5) denotes the final pseudospin direction at each *k*=1…*N*_*φ*_. The string {**n**(*k*)}_*k*_ of *N*_*φ*_ unit vectors can be viewed as a set of ‘classical' spins on a one-dimensional (1D) chain whose sites are labelled by the discrete index *k*, as in Fig. 1.

In the same notation,


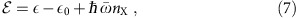


where


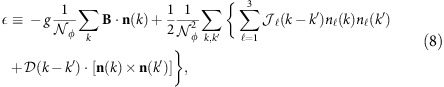



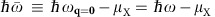
 and


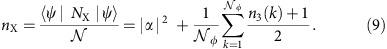


In equation (8), the quantity 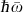 plays the role of a Lagrange multiplier, ɛ_0_ is a reference energy, which is defined, so that that =0 when *n*_X_=0, and 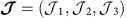 and 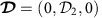 are the symmetric and antisymmetric, that is, Dzyaloshinsky–Moriya (DM)^41^,^42^, interactions between Bloch pseudospins. Explicit expression for 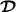, 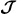 and ɛ_0_ are provided in Supplementary Note 4, together with plots of the Fourier transforms 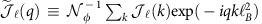 and 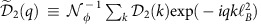 in Supplementary Fig. 2. In equation (9), we note a photon contribution *n*_ph_=〈*ψ*|*N*_ph_|*ψ*〉/*N*=|*α*|^2^ and an exciton contribution, 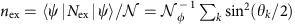. It is somewhat surprising that DM interactions appear in our energy functional (7), since these require spin-orbit interactions and appear when inversion symmetry is broken. Our microscopic Hamiltonian (1) does not contain either SOIs or breaks inversion symmetry. In the next section, we discuss the origin of pseudospin DM interactions.

Each of the terms in the expression (8) for *ɛ*=*ɛ*({*φ*_*k*_},{*θ*_*k*_},*α*) has a clear physical interpretation. The first term on the right-hand side is the energy of a set of independent 1D Bloch pseudospins in an effective magnetic field with the usual Rabi coupling and detuning contributions


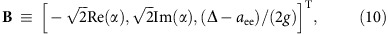


where Δ≡ℏ*ω*−(Ω_*M*_+Δ_ee_) is the detuning energy with Δ_ee_ a correction due to e–e interactions between electrons in the *M*,*M*+1 doublet and electrons in remote occupied LLs^43^,^44^,^45^ (see Supplementary Note 4). Because the MDF model applies over a large but *finite* energy interval, we need to introduce an ultraviolet cutoff *n*_max_ on the LL labels *n* of occupied states with *λ*=−1. Our choice for *n*_max_ is explained in the Supplementary Note 5. It is easy to demonstrate that Δ_ee_ depends logarithmically on *n*_max_: Δ_ee_=(*α*_ee_Ω_*M*_/8)[ln(*n*_max_)+*C*_*M*_] where *α*_ee_=*e*^2^/(*κ*_r_ℏ*v*_D_) is the graphene fine-structure constant^46^ and *C*_*M*_ is an ultraviolet-finite constant. For *M*=1 we find that 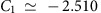, in agreement with earlier work^45^. The correction Δ_ee_ to the cyclotron transition energy is related to the extensively studied^47^,^48^,^49^ renormalization of the Dirac velocity *v*_D_ due to exchange interactions which also occurs in the absence of a magnetic field. The quantity *a*_ee_ involves only e–e interactions within the *M*,*M* + 1 doublet (see Supplementary Note 4). For *M*=1 we find 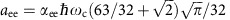. The second term in equation (8) describes interactions between Bloch pseudospins, which originate microscopically from matter-coherence dependence in the e–e interaction energy. At long wavelength these interactions stiffen the polariton condensate collective mode dispersion and support superfluidity. In the absence of a magnetic field their role at shorter wavelengths is masked by increasing exciton kinetic energy^50^.

### Pseudospin DM interactions

In the QHPF exciton fluid kinetic energy is quenched and, as we explain below, DM exciton–exciton interactions play an essential role in the physics. We therefore need to understand why 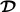 is finite. We start by observing (see Supplementary Note 4) that 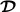 contains direct 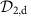 and exchange 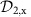 contributions, which (a) are of the same order of magnitude and (b) have the same sign. We can therefore focus on the direct contribution, which has a simple physical interpretation as the electrostatic interaction between two charge distributions that are uniform along the 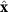 direction and vary along the 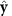 direction, that is,


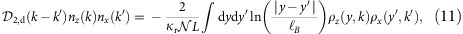


where


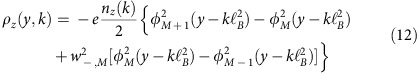


and


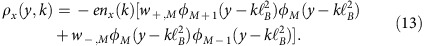


Here *φ*_*n*_(*y*) with *n*=0,1,2,… are normalized eigenfunctions of a 1D harmonic oscillator with frequency *ω*_c_ and 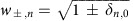 captures the property that the pseudospinor corresponding to the *n*=0 LL has weight only on one sublattice^36^.

We now use a multipole expansion argument to explain why 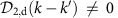. We first note that *ρ*_*z*_(*y*,*k*) has zero electrical monopole and dipole moments but finite quadrupole moment 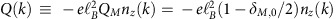. On the other hand, *ρ*_*x*_(*y*,*k*) has zero electrical monopole but finite dipole moment 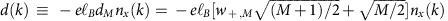. Using a multipole expansion, it follows that the leading contribution to equation (11) is the electrostatic interaction between a line of dipole moments extended along the *x*-direction and centred at one guiding centre and a line of quadrupole moments centred on the other guiding centre. It follows:


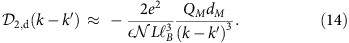


The interactions are antisymmetric, that is, their sign depends on whether the dipole is to the right or to the left of the quadrupole. The direct contributions between like pseudospin components which contribute to 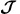 are symmetric because they are interactions between quadrupoles and quadrupoles or dipoles and dipoles.

Alert readers will have noted that only the 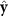-direction DM interaction is non-zero, 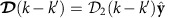. In contrast, the usual DM interaction^41^,^42^ is invariant under simultaneous rotation of orbital and spin degrees of freedom. This is not the case for pseudospin DM interactions: the property that only the component of 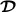 is non-zero can be traced to the property that, for a given sign of pseudospin *n*_*x*_(*k*), the charge distribution *ρ*_*x*_(*y*,*k*) in equation (13) changes sign under inversion around the guiding centre (that is, 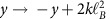).

### Linear stability analysis of the uniform fluid state

We first assume that the energy functional is minimized when *θ*_*k*_ and *φ*_*k*_ in equation (3) are *k*-‘independent', that is, *θ*_*k*_=*θ* and *φ*_*k*_=*φ* for every *k*. The functional then simplifies to


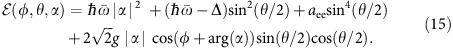


The first term on the right-hand side of equation (15), which is proportional to |*α*|^2^, is the free photon energy measured from the chemical potential *μ*_X_. The second term, which is proportional to sin^2^(*θ*/2), is the free exciton energy (as renormalized by e–e interactions, which enter in the definition of Δ). The third term, which is proportional to sin^4^(*θ*/2), is the exciton–exciton interaction term. Finally, the term in the second line, which is proportional to the Rabi coupling 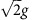, describes exciton–photon interactions.

We seek for a solution of the variational problem 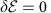 characterized by non-zero exciton and photon densities. For this to happen, the common chemical potential *μ*_X_ needs to satisfy the following inequality:


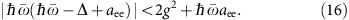


When this condition is satisfied, the solution of 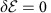 is given by


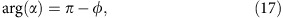



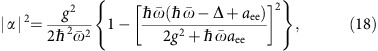


and


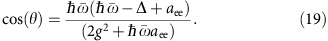


The common chemical potential *μ*_X_ must be adjusted to satisfy *n*_X_=[1−cos(*θ*)]/2+|*α*|^2^. In the spin-chain language introduced above this state is a collinear ferromagnet in which all the classical spins {**n**(*k*)}_*k*_ are oriented along the same direction, as in Fig. 1a. Note that, as expected, the energy minimization problem does not determine the overall phase of the condensate.

We now carry out a local stability analysis to understand what is the region of parameter space in which this polariton state is a local energy minimum. A minimum of 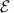, subject to the constraint on the average density *n*_X_ of excitations, is also a minimum of the functional ɛ({*φ*_*k*_},{*θ*_*k*_},*α*) defined in equation (8) with |*α*| not considered as an independent variable but rather viewed as a function of the variational parameters {*θ*_*k*_} through the use of equation (9), that is, with 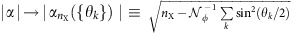. With this replacement,


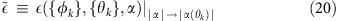


becomes a functional of 2*N*_*φ*_+1 independent variational parameters, which can be arranged, for the sake of simplicity, into a vector *w* with components 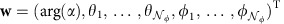.

In this notation, the extremum discussed above can be represented by the vector **w**_0_=(*π*−*φ*,*θ*,…,*θ*,*φ*,…,*φ*)^T^. We have checked that **w**_0_ is a solution of the equation 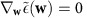. Whether **w**_0_ is a local minimum or maximum depends on the spectrum of the Hessian


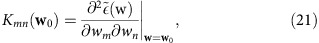


which is a (2*N*_*φ*_+1) × (2*N*_*φ*_+1) symmetric matrix.

**Figure 2:**
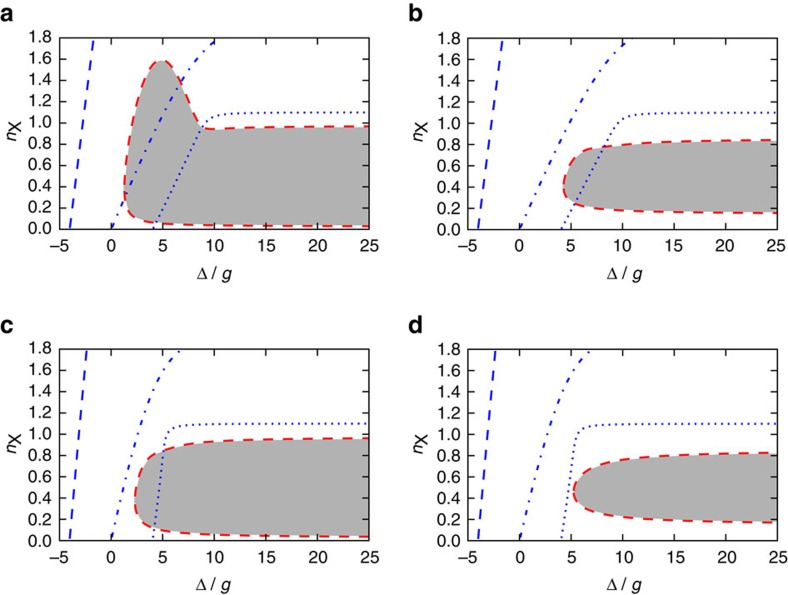
Phase diagram of a graphene integer QHPF. White (grey-shaded) regions represent the values of the detuning Δ—in units of *g*—and density *n*_X_ of total excitations at which the homogeneous phase described by equations (17, 18, 19) is stable (unstable). (**a**) *κ*_r_=5 and *M*=1; (**b**) *κ*_r_=5 and *M*=2; (**c**) *κ*_r_=15 and *M*=1; and (**d**) *κ*_r_=15 and *M*=2. In each panel, blue lines denote the location of points in the plane (Δ/*g*,*n*_X_), where the ratio between the number of excitons and the number of photons is constant: *n*_ex_/*n*_ph_=1/10 (dashed line), *n*_ex_/*n*_ph_=1 (dash-dotted line) and *n*_ex_/*n*_ph_=10 (dotted line). These curves have been calculated with reference to the homogenous phase.

The homogeneous polariton fluid phase is stable only if *K*_*mn*_(**w**_0_) has no negative eigenvalues. The stability analysis is simplified by exploiting translational symmetry to classify state fluctuations by momentum. Stability phase diagrams for *M*=1 and *M*=2, constructed by applying this criterion, are plotted in Fig. 2 for two different values of the cavity dielectric constant *κ*_r_. In this figure, white (grey-shaded) regions represent the values of the detuning Δ and density *n*_X_ of total excitations for which the homogeneous fluid phase is stable (unstable). As expected, by increasing *κ*_r_ (that is, reducing the importance of e–e interactions) the stable regions expand at the expense of the unstable ones. Note that the instability displays an intriguing ‘re-entrant' character and that it can occur also when matter and light have comparable weight, that is, when *n*_ex_∼*n*_ph_.

We have checked that the root of instability of the homogeneous fluid phase is e–e interactions. More precisely, it is possible to see that in the absence of DM interactions—that is, when 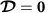 in equation (8)—the instability disappears. Symmetric interactions, however, still play an important quantitative role in the phase diagrams, as explained in Supplementary Note 6. The physics of these phase diagrams is discussed further below where we identify the phase diagram boundary with the appearance of soft-modes in the uniform polariton fluid collective mode spectrum. Stable phases occur only if 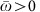 (that is, 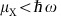). We remind the reader that this condition on *μ*_X_ is additional to the one given in equation (16) above.

### Elementary excitations of the polariton fluid

We evaluate the elementary excitations of the uniform polariton fluid^1^,^2^,^3^ by linearizing the Heisenberg coupled equations of motion of the matter Bloch pseudospin and photon operators using a Hartree–Fock factorization for the e–e interaction term in the Hamiltonian (see Supplementary Note 7). The collective excitation energies diagonalize the matrix


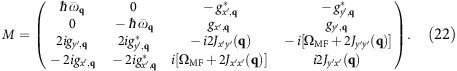


The first two-components of eigenvectors of *M* correspond to photon creation and annihilation, and the third and fourth to rotations of the Bloch pseudospin in a plane (denoted by 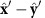 in the Supplementary Note 7) orthogonal to its ground state orientation. In equation (22) 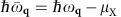, 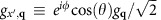, 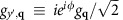,


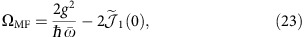



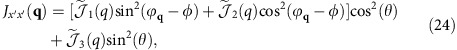



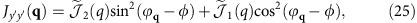



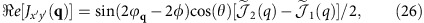



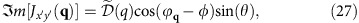


and **q**=[*q* cos(*ϕ*_**q**_), *q* sin(*ϕ*_**q**_)]^T^.

**Figure 3:**
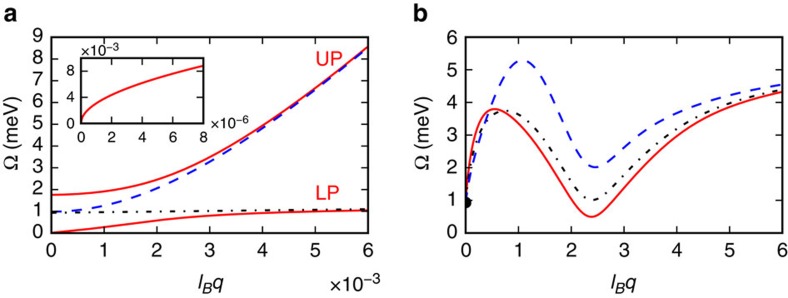
Collective excitation spectrum of the homogeneous fluid phase. (**a**) Dispersion relations of upper and lower dressed polariton modes (solid lines) in the long-wavelength 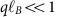 limit. The dashed line (dash-dotted line) represents the cavity photon dispersion (bright electronic collective mode), when the electron–photon coupling *g*_*q*_ is set to zero. In this panel *ϕ*_**q**_=*φ*. The inset shows a zoom of the lower polariton dispersion relation for 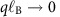. Note that the dispersion behaves as (*q*)^1/2^ in this limit. (**b**) Dispersion relation of the lower polariton mode, along three directions: *ϕ*_**q**_=*φ* (solid line), *ϕ*_**q**_=*φ*+*π*/4 (dash-dotted line) and *ϕ*_**q**_=*φ*+*π*/2 (dashed line). All the data in this figure have been obtained by setting *κ*_r_=5, *B*=0.5 Tesla, *M*=1, *n*_X_=0.1 and Δ=*g*.

The solution of the eigenvalue problem yields two hybrid modes that can be viewed as lower polaritons (LP) and upper polaritons (UP) that are dressed by the condensate and have strong mixing between photon and matter degrees of freedom at 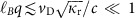. Figure 3 illustrates the dispersion relations of these two modes for *M*=1. For wavelengths comparable to the magnetic length, 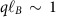, the UP mode has nearly pure photonic character, while the LP mode is a nearly pure matter excitation with a dispersion relation that is familiar from the theory of magnon energies in systems with asymmetric DM exchange interactions^51^:


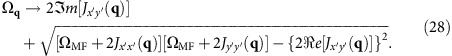


Figure 3b shows the LP dispersion relation for three different polar angles *ϕ*_**q**_. In all cases, a local roton-like minimum occurs at a wave vector 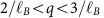. The global minimum of the LP dispersion occurs along the direction *ϕ*_**q**_=*φ*, where the impact of DM interactions is strongest, that is, 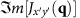 is maximum—see equation (27). The mode energy vanishes, and a Hessian eigenvalue crosses from positive to negative signalling instability, when


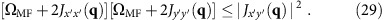


Since the LP mode becomes unstable at a ‘finite' wave vector 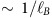, we conclude that the true ground state spontaneously breaks translational symmetry. We emphasize that softening of collective modes in quantum Hall fluids can be experimentally studied, for example, by inelastic light scattering^52^.

### Modulated phase of QHPFs

Motivated by the properties of magnetic systems with strong asymmetric spin interactions^51^, we seek broken translational states in which the Bloch pseudospins execute a small amplitude spiral around a mean orientation, as in Fig. 1b. This is a state in which *θ*_*k*_,*φ*_*k*_ have a rather simple *k*-dependence of the form:


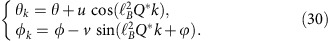


Equation (30) physically describes a small-amplitude spatially periodic contribution to the uniform condensate state (3) with *θ*_*k*_=*θ* and *φ*_*k*_=*φ*. One should therefore not confuse the condensed state described by equations (3 and 30) with a uniform condensate, in which electrons and holes form pairs with a finite centre-of-mass momentum^53^.

Because the form factors of electrons in *M* and *M*+1 LLs differ, this state has non-uniform electron charge density with periodicity 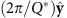. The Fourier transform of the density variation


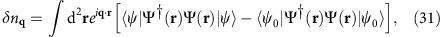


is non-zero only for **q**=(0, nQ*), where *n* is a relative integer. In equation (31) Ψ(**r**)=∑_λ,*n*,*k*,*ξ*_〈**r**|λ,*n*,*k*〉*c*_λ,*n*,*k*,*ξ*_ with


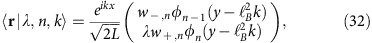


is a field operator that creates an electron at position **r** (ref. 36).

**Figure 4:**
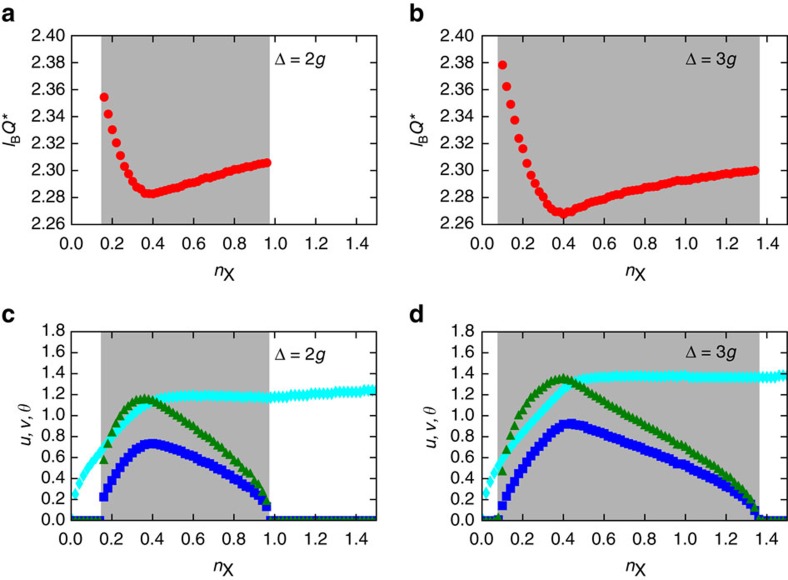
Variational parameters of the modulated phase. This figure shows the optimal values of the variational parameters *u*,*v*,*θ* and Q* in equation (30) for a cavity dielectric constant *κ*_r_=5, highest occupied LL *M*=1 and different values of the detuning Δ. (**a**,**c**) Δ=2*g*. (**b**,**d**) Δ=3*g*. (**a**,**b**) Dependence of the characteristic wave number Q* (in units of 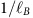) on the density *n*_X_ of total excitations. (**c**,**d**) Dependence of the quantities *u* (blue squares), *v* (green triangles) and *θ* (cyan diamonds) on the density *n*_X_ of total excitations. Grey-shaded areas have the same meaning as in Fig. 2.

**Figure 5:**
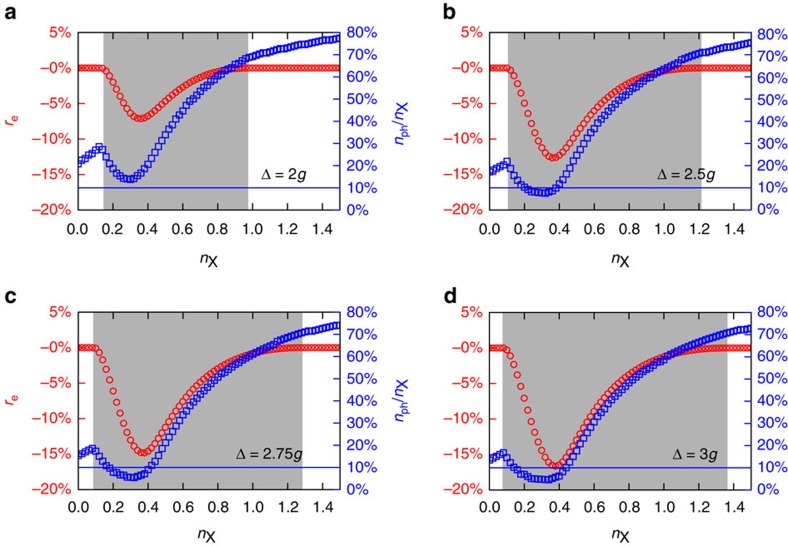
Energetics and photon densities in the modulated phase. Data denoted by red circles represent the quantity *r*_e_ in equation (33) plotted as a function of *n*_X_. Data denoted by blue squares represent the ratio between the photon density *n*_ph_ and the density *n*_X_ of total excitations, as a function of *n*_X_ and for the modulated phase only. Data in this figure have been obtained by setting the dielectric constant at *κ*_r_=5 and the highest occupied LL at *M*=1. Above the horizontal blue line, *n*_ph_/*n*_X_>10%. Different panels refer to different values of the ratio Δ/*g*: (**a**) Δ/*g*=2; (**b**) 2.5; (**c**) 2.75; and (**d**) 3.

For this form of variational wave function we have fixed *θ*, *φ*, *α*, *u*, *v*, Q* and *ϕ* by minimizing 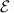. A summary of our main numerical results for *u*, *v*, *θ* and Q* is reported in Fig. 4 for two values of the detuning Δ. Minimization yields *ϕ*=0, *φ*=−*π*/2 and arg(*α*)=*π*−*φ*. The dependence of |*α*| on *n*_X_ is given by 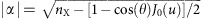, where *J*_0_(*x*) is the Bessel function of order zero. Fig. 4a,b illustrate the weak dependence of the characteristic wave number Q* on the density *n*_X_ of total excitations. In Fig. 5, we report, for each value of *n*_X_, the ratio


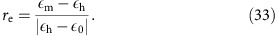


The numerator in equation (33) is the difference between the energy of the condensed modulated phase, *ɛ*_m_, described by equation (30), and that of the condensed homogeneous phase, ɛ_h_, described by equations (17, 18, 19). In the condensed modulated phase, it follows that *r*_e_<0. The denominator in equation (33) is the condensate energy in the homogeneous phase. In Fig. 5 we clearly see that, depending on the detuning Δ and the density of excitations *n*_X_, the modulated phase comes with a condensate energy gain *r*_e_ in the window ≈ 5–15%, with values of the photon fraction that are well above 10%.

## Discussion

In this article, we have made two major simplifying approximations that deserve a detailed discussion. We have (i) used a quasi-equilibrium approach based on a grand-canonical Hamiltonian and (ii) treated e–e interactions at the mean-field level.

(i) Exciton-polariton condensates differ from ultracold atomic gases in that the condensing quasi-particles have relatively short lifetimes, mainly because of photon losses in the cavity or metamaterial. External optical pumping is therefore needed to maintain a non-equilibrium steady state. It has been shown^54^ that the resulting non-equilibrium steady state can be approximated by a thermal equilibrium state when the thermalization time is shorter than the exciton-polariton lifetime. Equilibrium approximations have been successfully used in the literature to describe exciton-polariton fluids in semiconductor microcavities^2^,^38^,^39^,^55^,^56^. Experimental studies in GaAs quantum wells have shown that the thermalization time criterion is satisfied above a critical pump level^57^ and that polariton–polariton interactions (which are responsible for thermalization) are strong^58^. We assume below that a similar thermal equilibrium state can be achieved in graphene QHPFs. Because polaritons interact more strongly when they have a larger excitation fraction, quasi-equilibrium polariton condensates are expected to be more accessible experimentally when the cavity photon energy is higher than the bare exciton energy, that is, at positive detuning.

(ii) The possibility of non-mean-field behaviour in the matter degrees of freedom is an issue. Mean-field-theory is accurate for dilute excitons at low temperatures^59^, but could fail at high exciton densities. In particular, the modulated phase we have found may undergo quantum melting. However, matter degrees of freedom at integer filling factors in the quantum Hall regime tend to be often well described by mean-field theory^37^. The accuracy of mean-field theory is generally related to the restricted Hilbert space of LLs, which preclude the formation of competing correlated states with larger quantum fluctuations. There are several examples of interesting broken symmetry states in both semiconductor quantum wells and graphene that are accurately described by mean-field theory, including spin-polarized ferromagnetic states at odd filling factors^60^, coherent quantum Hall bilayers in semiconductors systems with coupled quantum wells^61^ and spin-density wave states in neutral graphene^36^. In some cases, the state selected by mean-field-theory energy minimization is the only state in the quantum Hall Hilbert space with a given set of quantum numbers, and therefore is exact. The situation here is similar to the coherent bilayer state^61^ in that we have coherence between adjacent LLs.

Finally, we mention that physics similar to that described in this article is not expected to be limited to graphene but should equally occur in 2D electron gases in semiconductor (for example, GaAs) quantum wells. There are a number of quantitative differences in detail, however. Most critically, the anharmonic LL spectrum of graphene should make it possible to achieve a better selective coupling to a particular *M*,*M*+1 doublet^25^.

### Data availability

The data files used to prepare the figures shown in the manuscript are available from the corresponding author upon request.

## Additional information

**How to cite this article:** Pellegrino, F. M. D. *et al.* Modulated phases of graphene quantum Hall polariton fluids. *Nat. Commun.* **7,** 13355 doi: 10.1038/ncomms13355 (2016).

**Publisher's note:** Springer Nature remains neutral with regard to jurisdictional claims in published maps and institutional affiliations.

## Supplementary Material

Supplementary Information Supplementary Figures 1-3, Supplementary Notes 1-7 and Supplementary References.
