# Supplementary material for: Modulated phases of graphene quantum Hall polariton fluids
Source: Nat Commun. 2016 Nov 14;7:13355. doi: 10.1038/ncomms13355 (PMC5114533; doi:10.1038/ncomms13355)
Supplement: Supplementary Information — Supplementary Figures 1-3, Supplementary Notes 1-7 and Supplementary References. [file ncomms13355-s1.pdf]

Supplementary Figures

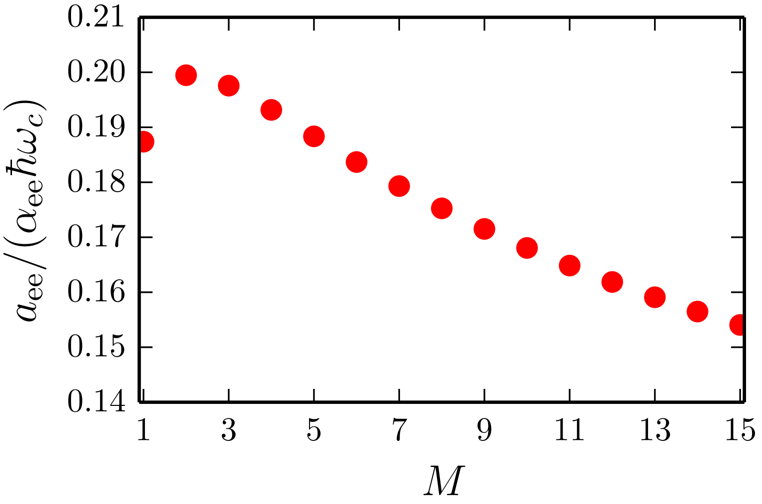

Supplementary Figure 1: **Dimensionless strength of electron-electron interactions.** Dependence of a_{ee} (in units of $\alpha_{\text{ee}}\hbar\omega_{\text{c}}$) on the Landau level index M.

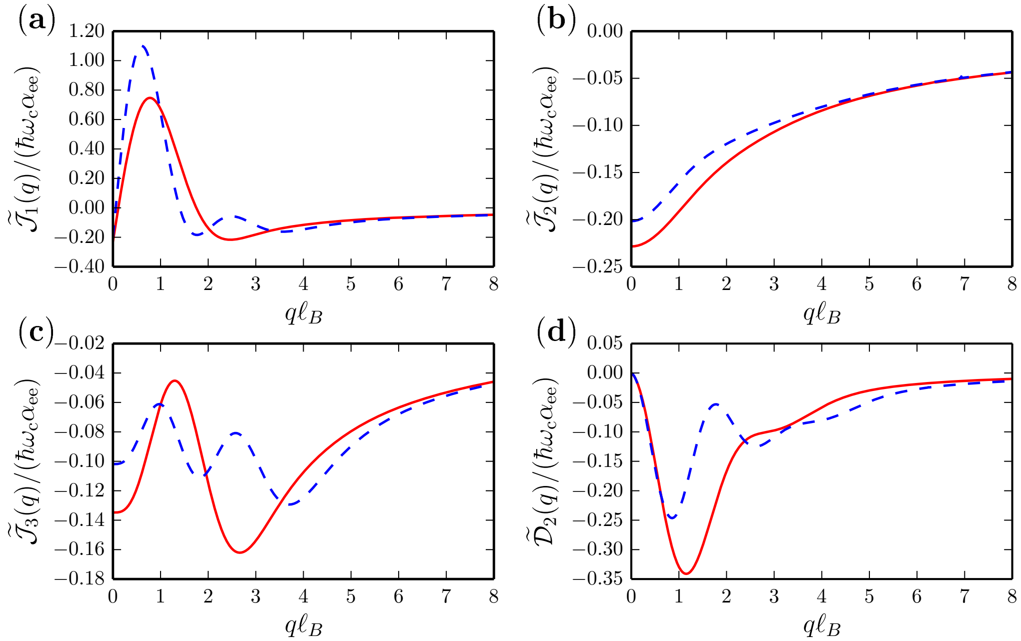

Supplementary Figure 2: **Pseudospin-pseudospin interactions.** Fourier transforms $\widetilde{\mathcal{J}}_\ell(q)$ and $\widetilde{\mathcal{D}}_2(q)$ (in units of $\hbar\omega_c\alpha_{ee}$). Panels (**a**), (**b**) and (**c**) refer to the symmetric interactions, while panel (**d**) refers to the antisymmetric interaction. The red solid (blue dashed) line refers to the case with highest occupied LL $M = 1$ ($M = 2$) in conduction band $\lambda = +$.

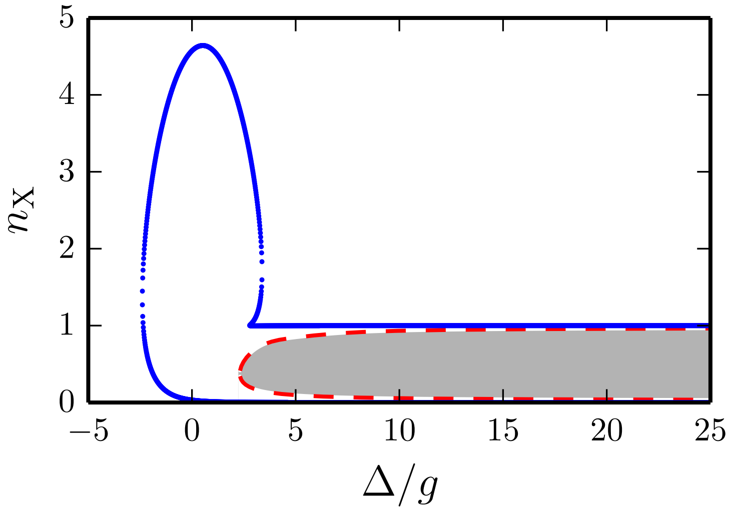

Supplementary Figure 3: **Phase diagram in the absence of symmetric interactions.** The red dashed boundary of the grey-shaded area—which represents the unstable region for $\mathcal{J}_\ell \neq 0$ as in Figure 2(**c**) of the main text—enlarges: for $\mathcal{J}_\ell = 0$ (absence of symmetric interactions) the new boundary is represented by the blue filled circles.

Supplementary Note 1

At low energies, charge carriers in graphene are modeled by the usual single-channel massless Dirac fermion (MDF) Hamiltonian [1, 2]

$$\mathcal{H}_{\rm D} = v_{\rm D}\sigma \cdot \mathbf{p} \ , \tag{1}$$

where $v_{\rm D} \approx 10^6$ m/s is the Dirac velocity. Here $\sigma = (\sigma_x, \sigma_y)$ is a 2D vector of Pauli matrices acting on sublattice degrees-of-freedom and $\mathbf{p} = -i\hbar\nabla_{\mathbf{r}}$ is the 2D momentum measured from one of the two corners (valleys) of the Brillouin zone.

A quantizing magnetic field $\mathbf{B} = B\hat{\mathbf{z}}$ perpendicular to the graphene sheet is coupled to the electronic degrees-of-freedom by replacing the canonical momentum $\mathbf{p}$ in Eq. (1) with the kinetic momentum $\mathbf{\Pi} = \mathbf{p} + e\mathbf{A}_0/c$, where $\mathbf{A}_0$ is the vector potential that describes the static magnetic field $\mathbf{B}$. The corresponding Hamiltonian is

$$\mathcal{H}_0 = v_{\rm D}\sigma \cdot \mathbf{\Pi} \ . \tag{2}$$

We work in the Landau gauge $\mathbf{A}_0 = -By\hat{\mathbf{x}}$. In this gauge the canonical momentum along the $\hat{\mathbf{x}}$ direction, p_x, coincides with the magnetic translation operator [3] along the same direction and it commutes with the Hamiltonian $\mathcal{H}_0$. Thus, the eigenvalues of p_x are good quantum numbers. A complete set of eigenfunctions of the Hamiltonian $\mathcal{H}_0$ in Eq. (2) is provided by the two component pseudospinors [4]

$$\langle \mathbf{r}|\lambda, n, k\rangle = \frac{e^{ikx}}{\sqrt{2L}} \left(\begin{array}{c} w_{-,n}\phi_{n-1}(y - \ell_B^2 k) \\ \lambda w_{+,n}\phi_n(y - \ell_B^2 k) \end{array} \right) \ , \tag{3}$$

where $\lambda = +$ $(-)$ denotes conduction (valence) band levels, $n \in \mathbb{N}$ is the Landau level (LL) index, and k is the eigenvalue of the magnetic translation operator in the $\hat{\mathbf{x}}$ direction. In Eq. (3)

$$w_{\pm,n} = \sqrt{1 \pm \delta_{n,0}} \tag{4}$$

guarantees that the pseudospinor corresponding to the $n = 0$ LL has nonzero weight only on one sublattice. Furthermore, $\phi_n(y)$ with $n = 0, 1, 2, \ldots$ are the normalized eigenfunctions of a 1D harmonic oscillator with frequency equal to the MDF cyclotron frequency $\omega_{\rm c} = \sqrt{2}v_{\rm D}/\ell_B$. Here $\ell_B = \sqrt{\hbar c/(eB)} \simeq$ 25 nm$/\sqrt{B[\rm Tesla]}$ is the magnetic length.

The spectrum of the Hamiltonian (2) has the well-known form [4]

$$\varepsilon_{\lambda,n} = \lambda\hbar\omega_{\rm c}\sqrt{n}\ . \tag{5}$$

Each LL has a macroscopic degeneracy $\mathcal{N} = N_{\rm f}S/(2\pi\ell_B^2) \equiv N_{\rm f}\mathcal{N}_\phi$, where $N_{\rm f} = 4$ is the spin-valley degeneracy and $S = L^2$ is the sample area.

The fully microscopic matter Hamiltonian is written as

$$\mathcal{H}_{\rm mat} = \mathcal{H}_0 + \mathcal{H}_{\rm ee}\ . \tag{6}$$

Here, $\mathcal{H}_0$ is the second-quantized version of Eq. (2),

$$\mathcal{H}_0 = \sum_{\lambda,n,k,\xi} \varepsilon_{\lambda,n} c^\dagger_{\lambda,n,k,\xi} c_{\lambda,n,k,\xi}\ , \tag{7}$$

where $c^\dagger_{\lambda,n,k,\xi}$ ($c_{\lambda,n,k,\xi}$) is a fermionic creation (annihilation) operator for an electron with band index λ, LL quantum number n, and eigenvalue of the magnetic translation operator along the $\hat{\mathbf{x}}$ direction equal to k. The collective index ξ refers to the valley (K, K') index and spin-projection along the $\hat{\mathbf{z}}$ direction. The second term in Eq. (6), $\mathcal{H}_{\rm ee}$, represents Coloumb interactions. This term can be written as

$$\mathcal{H}_{\rm ee} = \frac{1}{2L^2}\sum_{\mathbf{q}} v_{\mathbf{q}}\left[n_{-\mathbf{q}}n_{\mathbf{q}} - n_{\mathbf{q}=\mathbf{0}}\right]\ , \tag{8}$$

where $n_{\mathbf{q}}$ is the Fourier transform of the electronic density operator

$$n_{\mathbf{q}} \equiv \int d\mathbf{r} e^{-i\mathbf{q}\cdot\mathbf{r}}\psi^\dagger(\mathbf{r})\psi(\mathbf{r})\ , \tag{9}$$

and $v_{\mathbf{q}}$ is the 2D Fourier transform of the Coulomb potential

$$v_{\mathbf{q}} = \frac{2\pi e^2}{\epsilon q}\ . \tag{10}$$

Here $\kappa_{\rm r}$ is the cavity dielectric constant. The real-space field operators $\psi^\dagger(\mathbf{r})$ and $\psi(\mathbf{r})$ have been introduced in the main text.

Selecting only terms that respect the SU(4) spin-valley symmetry, the e-e interaction term in Eq. (8) can be written in the following manner [4]

$$\begin{aligned}\mathcal{H}_{\rm ee} &= \frac{1}{2}\frac{1}{L^2}\sum_{\mathbf{q}}\sum_{\lambda_i,n_i}\sum_{\xi,\xi',k,k'} e^{i\ell_B^2 q_y(k-k')}\mathcal{V}_{(\lambda_1 n_1),(\lambda_2 n_2),(\lambda_3 n_3),(\lambda_4 n_4)}(\mathbf{q}) \\ &\times\ c^\dagger_{\lambda_1,n_1,\xi,k}c^\dagger_{\lambda_2,n_2,\xi',k'-q_x}c_{\lambda_3,n_3,\xi',k'}c_{\lambda_4,n_4,\xi,k-q_x}\ ,\end{aligned} \tag{11}$$

where

$$\mathcal{V}_{(\lambda_1,n_1),(\lambda_2,n_2),(\lambda_3,n_3),(\lambda_4,n_4)}(\mathbf{q}) = v_{\mathbf{q}}\mathcal{F}_{(\lambda_1,n_1),(\lambda_4,n_4)}(-\mathbf{q})\mathcal{F}_{(\lambda_2,n_2),(\lambda_3,n_3)}(\mathbf{q}) \ . \quad (12)$$

The form factors $\mathcal{F}_{(\lambda,n),(\lambda',n')}(\mathbf{q})$ are given by [4]

$$\begin{aligned}\mathcal{F}_{(\lambda,n),(\lambda',n')}(\mathbf{q}) \ &\equiv \ \frac{1}{2}\Bigg[w_{-,n}w_{-,n'}D_{n-1,n'-1}\left(-\frac{\ell_B\bar{q}^*}{\sqrt{2}}\right) \\ &+ \ \lambda\lambda' w_{+,n}w_{+,n'}D_{n,n'}\left(-\frac{\ell_B\bar{q}^*}{\sqrt{2}}\right)\Bigg] \ . \end{aligned} \quad (13)$$

Here, $\bar{q} = q_x + iq_y$ and $\bar{q}^* = q_x - iq_y$,

$$D_{n,n'}(z) \equiv \begin{cases} \sqrt{\dfrac{n'!}{n!}}z^{n-n'}e^{-|z|^2/2}L_{n'}^{(n-n')}(|z|^2), & \text{for } n \geq n' \\ D_{n',n}(-z^*), & \text{for } n < n' \end{cases} \ , \quad (14)$$

and $L_n^{(n-n')}(x)$ are generalized Laguerre polynomials [5].

We consider the integer quantum Hall regime in which a given number of LLs are fully occupied and the remaining ones are empty. Since the MDF Hamiltonian is particle-hole symmetric, we can consider, without loss of generality, the situation in which graphene is n-doped and the Fermi energy lies in the conduction band ($\lambda = +$). We denote by $n = M$ the highest occupied LL, and the lowest empty LL is $n = M + 1$ We are interested in the case in which cavity photons with energy $\hbar\omega$ are nearly resonant with the energy difference between the two conduction-band LLs $n = M, M+1$. In this limit, the fermionic Hilbert space can be reduced to the resonant doublet. From here on, we denote by $\tilde{c}_{\lambda,n,k,\xi}$ and $\tilde{c}^\dagger_{\lambda,n,k,\xi}$ operators for states which do not belong to the resonant doublet $M, M + 1$. We will keep using $c_{\lambda,n,k,\xi}$ and $c^\dagger_{\lambda,n,k,\xi}$ only for states which belong to the resonant doublet. We can rewrite the full microscopic Hamiltonian in Eq. (6) as the sum of three terms:

$$\mathcal{H}_{\rm mat} = \mathcal{H}_{\rm d} + \mathcal{H}_{\rm m} + \mathcal{H}_{\rm dm} \ . \quad (15)$$

The first term $\mathcal{H}_{\rm d}$ contains only fermionic field operators related to the resonant doublet $M, M+1$. The second term, $\mathcal{H}_{\rm m}$, contains only field operators of the type $\tilde{c}_{\lambda,n,k,\xi}$, $\tilde{c}^\dagger_{\lambda,n,k,\xi}$: these degrees of freedom play the role of a "medium" for the resonant doublet. The third term, $\mathcal{H}_{\rm dm}$, describes coupling between the resonant doublet and the medium degrees of freedom.

To obtain an *effective* matter Hamiltonian, we start from the fully microscopic Hamiltonian $\mathcal{H}_{\rm mat}$ and we treat in an exact fashion all the terms that involve field operators ($c_{\lambda,n,k,\xi}$ and $c^\dagger_{\lambda,n,k,\xi}$) acting only on the doublet $n = M, M+1$ in conduction band $\lambda = +$. All the other terms (containing $\tilde{c}_{\lambda,n,k,\xi}$ and $\tilde{c}^\dagger_{\lambda,n,k,\xi}$) are treated within the Hartree-Fock approximation [3]. The medium Hamiltonian is discarded. In the coupling term $\mathcal{H}_{\rm dm}$, we only keep terms that separately conserve the number of particles in the $M, M+1$ doublet and in the medium. These are terms of the form $\tilde{c}^\dagger c^\dagger c\tilde{c}$. Terms of the form $\tilde{c}^\dagger\tilde{c}^\dagger cc$ are discarded. We therefore replace

$$\begin{aligned}&\tilde{c}^\dagger_{\lambda_1,n_1,k_1,\xi_1} c^\dagger_{+,n_2,k_2,\xi_2} c_{+,n_3,k_3,\xi_3} \tilde{c}_{\lambda_4,n_4,k_4,\xi_4} \longrightarrow \\ &\delta_{\lambda_1,\lambda_4}\delta_{n_1,n_4}\delta_{k_1,k_4}\delta_{\xi_1,\xi_4}\Theta(\epsilon_{+,M}-\epsilon_{\lambda_1,n_1}) c^\dagger_{+,n_2,k_2,\xi_2} c_{+,n_3,k_3,\xi_3}\ .\end{aligned} \tag{16}$$

After straightforward algebraic manipulations we reach the final result for the effective matter Hamiltonian, which is best expressed in a pseudospin representation in which pseudospin "up" ("down") corresponds to the $M+1$ (M) LL. To this end, we define the following pseudospin operators:

$$\bar{\rho}^{\xi\xi'}_m(\mathbf{q}) = \sqrt{\frac{1}{\mathcal{N}_\phi}} \sum_{n,n'=M,M+1} \sum_k [\tau_m]_{nn'} c^\dagger_{+,n,k,\xi} c_{+,n',k+q_x,\xi'}\ e^{-i\ell_B^2 q_y (k+q_x/2)}\ , \tag{17}$$

and

$$S_m(\mathbf{q}) = \sqrt{\frac{1}{N_{\rm f}}} \sum_\xi \bar{\rho}^{\xi\xi}_m(\mathbf{q})\ , \tag{18}$$

where $m = 0, \ldots, 3$ and $[\tau_m]_{nn'}$ labels the matrix elements of a four-vector τ_m of 2×2 matrices acting on the $M, M+1$ doublet, specifically τ_0 represents the 2×2 identity matrix and τ_1, τ_2, τ_3 represent the ordinary 2×2 Pauli matrices.

The final effective matter Hamiltonian reads as following:

$$\mathcal{H}_{\rm mat} = E^\star_M \sqrt{\mathcal{N}} S_0(0) + \frac{\Omega^\star_M}{2}\sqrt{\mathcal{N}} S_3(0) + \frac{1}{2}\sum_{m,m',\mathbf{q}} V_{mm'}(\mathbf{q}) S_m(-\mathbf{q}) S_{m'}(\mathbf{q})\ . \tag{19}$$

Here, $E^\star_M \equiv E_M + \tilde{E}_M$ and $\Omega^\star_M \equiv \Omega_M + \tilde{\Omega}_M$ with $E_M = \hbar\omega_{\rm c}(\sqrt{M+1} + \sqrt{M})/2$. The terms $\tilde{E}_M$ and $\tilde{\Omega}_M$ are due to exchange interactions between

the resonant doublet $M, M+1$ and *occupied* LLs outside the doublet, i.e.

$$\begin{aligned}\tilde{E}_M &= -\frac{1}{2L^2}\sum_{\mathbf{q}}\sum_{\lambda,n}{}' \Big[\mathcal{V}_{(+,M+1),(\lambda,n),(+,M+1),(\lambda,n)}(\mathbf{q}) \\ &+ \mathcal{V}_{(+,M),(\lambda,n),(+,M),(\lambda,n)}(\mathbf{q})\Big] \end{aligned} \tag{20}$$

and

$$\begin{aligned}\tilde{\Omega}_M &= -\frac{1}{2L^2}\sum_{\mathbf{q}}\sum_{\lambda,n}{}' \Big[\mathcal{V}_{(+,M+1),(\lambda,n),(+,M+1),(\lambda,n)}(\mathbf{q}) \\ &- \mathcal{V}_{(+,M),(\lambda,n),(+,M),(\lambda,n)}(\mathbf{q})\Big] , \end{aligned} \tag{21}$$

where the summation over λ, n runs only values such that $\epsilon_{\lambda,n} < \epsilon_{+1,M}$.

The last term in Eq. (19) describes e-e interactions within the resonant doublet $M, M+1$, i.e.

$$V_{mm'}(\mathbf{q}) = \frac{1}{4}\sum_{n_i=M,M+1}[\tau_m]_{n_1,n_4}[\tau_{m'}]_{n_2,n_3}\mathcal{V}_{(+,n_1),(+,n_2),(+,n_3),(+,n_4)}(\mathbf{q}) . \tag{22}$$

This is a 4×4 Hermitian matrix, which can be decomposed into its real and imaginary parts: $V_{mm'}(\mathbf{q}) = \mathrm{Re}[V_{mm'}(\mathbf{q})] + i\mathrm{Im}[V_{mm'}(\mathbf{q})]$, where

$$\begin{cases} \mathrm{Re}[V_{mm'}(\mathbf{q})] = \mathrm{Re}[V_{m'm}(\mathbf{q})] \\ \mathrm{Im}[V_{mm'}(\mathbf{q})] = -\mathrm{Im}[V_{m'm}(\mathbf{q})] \end{cases} . \tag{23}$$

It is possible to show that every quantity $V_{mm'}(\mathbf{q})$ is either purely real or purely imaginary: $V_{mm'}(\mathbf{q})$ is purely imaginary if $m = 1$ or 2 ($m = 0$ or 3) and simultaneously $m' = 0$ or 3 ($m' = 1$ or 2); for any other value of m, m' $V_{mm'}(\mathbf{q})$ is purely real.

Supplementary Note 2

We consider a graphene sheet coupled to the electromagnetic field in a cavity. The Hamiltonian that describes the coupling between electrons and cavity

photons is

$$\mathcal{H}_{\rm int} = \frac{1}{\sqrt{\mathcal{N}}} \sum_{\lambda,\lambda',n,n',\xi,\xi',k,\mathbf{q},\nu} e^{i\ell_B^2 q_y(k+q_x/2)} g_{\mathbf{q}} \big[\lambda w_{\lambda n} e^{-}_{\rm em}(\mathbf{q},\nu)\delta_{n',n+1} + \lambda' w_{\lambda' n'} e^{+}_{\rm em}(\mathbf{q},\nu)\delta_{n',n-1}\big]\left(a_{\mathbf{q},\nu} + a^\dagger_{-\mathbf{q},\nu}\right) c^\dagger_{\lambda' n' k+q_x} c_{\lambda n k} ~, \qquad (24)$$

where $g_{\mathbf{q}} = \hbar\omega_{\rm c}\sqrt{e^2/(2\epsilon L_z \hbar\omega_{\mathbf{q}})}$ is the light-matter interaction parameter, $e^{\pm}_{\rm em}(\mathbf{q},\nu) = (\hat{\mathbf{x}} \pm i\hat{\mathbf{y}}) \cdot \mathbf{e}_{\rm em}(\mathbf{q},\nu)$ where $\mathbf{e}_{\rm em}(\mathbf{q},\nu)$ is a unit vector describing the linear polarization ν of the electromagnetic field, $\omega_{\mathbf{q}} = \sqrt{\omega^2 + c^2 q^2/\kappa_r}$ is the cavity photon dispersion relation, κ_r is the cavity dielectric constant, $V = L_z L^2$ the volume of the cavity, and $L_z \ll L$ is the cavity length along the $\hat{\mathbf{z}}$ direction.

Among all the processes described by $\mathcal{H}_{\rm int}$, we take into account only resonant terms which describe the photon-induced electronic transitions between LLs M and $M+1$ in conduction band. This approximation is called rotating wave approximation (RWA). In the RWA, the light-matter interaction Hamiltonian becomes

$$\mathcal{H}_{\rm int} = \sqrt{2}\sum_{\mathbf{q}} g_{\mathbf{q}} \left[a^\dagger_{\mathbf{q},{\rm L}} S_-(\mathbf{q}) + a_{-\mathbf{q},{\rm L}} S_+(\mathbf{q})\right] ~, \qquad (25)$$

where $a_{\mathbf{q},{\rm L}} = (a_{\mathbf{q},x} - i a_{\mathbf{q},y})/\sqrt{2}$ [$a^\dagger_{\mathbf{q},{\rm L}} = (a^\dagger_{\mathbf{q},x} + i a^\dagger_{\mathbf{q},y})/\sqrt{2}$] is the annihilation [creation] operator for a left-handed photon and $S_\pm(\mathbf{q}) \equiv [S_1(\mathbf{q}) \pm i S_2(\mathbf{q})]/2$. The term containing S_+ (S_-) describes transitions from LL M ($M+1$) to LL $M+1$ (M) assisted by the annihilation (creation) of a left-handed photon. For $\mathbf{B} = B\hat{\mathbf{z}}$, the RWA selects the left circular polarization, $\mathbf{e}_{\rm em}(\mathbf{q},{\rm L}) = (\hat{\mathbf{x}} - i\hat{\mathbf{y}})/\sqrt{2}$: of course, the RWA will select the right circular polarization, $\mathbf{e}_{\rm em}(\mathbf{q},{\rm R}) = (\hat{\mathbf{x}} + i\hat{\mathbf{y}})/\sqrt{2}$, for $\mathbf{B} = -B\hat{\mathbf{z}}$.

Within the RWA, the sum of the number of cavity photons $N_{\rm ph}$ and the number of excitons $N_{\rm ex}$ is a *conserved* quantity. The number of photons can be expressed in terms of the photon field operator as

$$N_{\rm ph} \equiv \sum_{\mathbf{q},\nu} a^\dagger_{\mathbf{q},\nu} a_{\mathbf{q},\nu} ~, \qquad (26)$$

while the number the number of excitons can be expressed in terms of the pseudospin operators as

$$N_{\rm ex} \equiv \mathcal{N}[1 + S_3(0)/\sqrt{\mathcal{N}}]/2 ~. \qquad (27)$$

Supplementary Note 3

It is possible to show that the variational state $|\psi\rangle$ introduced in Eq. (3) of the main text can be re-written as:

$$|\psi\rangle = e^{-\mathcal{N}\frac{|\gamma|^2}{2}} e^{\sqrt{\mathcal{N}}\gamma p^\dagger} |\psi_0\rangle \ , \tag{28}$$

where

$$p^\dagger \equiv \frac{1}{\gamma}\left(\alpha a^\dagger_{\mathbf{0},\mathrm{L}} + \beta d^\dagger\right) \ , \tag{29}$$

$$d^\dagger \equiv \frac{1}{\sqrt{\mathcal{N}}\beta}\sum_{k,\xi} e^{-i\phi_k}\tan(\theta_k/2) c^\dagger_{+,M+1,k,\xi} c_{+,M,k,\xi} \ , \tag{30}$$

$$\beta \equiv \sqrt{-\frac{2}{\mathcal{N}}\sum_{k,\xi}\log(\cos\theta_k/2)} \ , \tag{31}$$

and

$$\gamma \equiv \sqrt{|\alpha|^2 + |\beta|^2} \ . \tag{32}$$

We note that the commutator between the operator $d^\dagger$ and its hermitian conjugate d is given by:

$$[d, d^\dagger] = \frac{1}{\mathcal{N}\beta^2}\sum_{k,\xi}\tan^2(\theta_k/2)\left(c^\dagger_{+1,M,k,\xi}c_{+1,M,k,\xi} - c^\dagger_{+1,M+1,k,\xi}c_{+1,M+1,k,\xi}\right) \ . \tag{33}$$

Calculating the expectation value of $[d, d^\dagger]$ on the variational state introduced in Eq. (3) of the main text and taking the low-density limit $\theta_k \ll 1$, we find:

$$\langle\psi|[d, d^\dagger]|\psi\rangle \approx 1 - \frac{\sum_k \theta_k^4}{2\sum_k \theta_k^2} \ . \tag{34}$$

If the second term on the right-hand side of the previous equation is neglected, excitons can be treated as bosons. In the same limit, the operator $p^\dagger$ has the meaning of a polariton creation operator. The variational state reported in Eq. (3) of the main text is a coherent state of polaritons and the quantity $\mathcal{N}|\gamma|^2$ represents the average number of polaritons.

Supplementary Note 4

We here report a number of relevant parameters that appear in the energy functional introduced in Eq. (7) of the main text:

$$\Delta_{\rm ee} \equiv \frac{1}{L^2}\sum_{\mathbf{q}} v_{\mathbf{q}}\Big\{\sum_{\lambda,n}{}' \left[|\mathcal{F}_{(+,M),(\lambda,n)}(\mathbf{q})|^2 - |\mathcal{F}_{(+,M+1),(\lambda,n)}(\mathbf{q})|^2\right] - \mathcal{F}_{(+,M),(+,M)}(\mathbf{q})\mathcal{F}_{(+,M+1),(+,M+1)}(\mathbf{q})\Big\}~, \tag{35}$$

$$a_{\rm ee} \equiv \frac{1}{L^2}\sum_{\mathbf{q}} v_{\mathbf{q}}\Big\{-\frac{1}{2}\left[\mathcal{F}_{(+,M),(+,M)}(\mathbf{q}) - \mathcal{F}_{(+,M+1),(+,M+1)}(\mathbf{q})\right]^2 + |\mathcal{F}_{(+,M+1),(+,M)}(\mathbf{q})|^2\Big\}~, \tag{36}$$

and

$$\epsilon_0 \equiv \frac{\Delta - a_{\rm ee}}{2} - \frac{1}{8L^2}\sum_{\mathbf{q}} v_{\mathbf{q}}\big[2|\mathcal{F}_{(+,M+1),(+,M)}(\mathbf{q})|^2 - |\mathcal{F}_{(+,M+1),(+,M+1)}(\mathbf{q})|^2 - |\mathcal{F}_{(+,M),(+,M)}(\mathbf{q})|^2\big]~. \tag{37}$$

As in Supplementary Note 1, the sum over λ, n runs only over indices such that $\epsilon_{\lambda,n} < \epsilon_{+,M}$. We note that $a_{\rm ee}$ involves only the two resonant LLs M and $M+1$.

Supplementary Figure 1 shows the quantity $a_{\rm ee}$ (in units of $\alpha_{\rm ee}\hbar\omega_{\rm c}$) as a function of the LL index M in the interval $1 \leq M \leq 15$. Here $\alpha_{\rm ee} = e^2/(\kappa_{\rm r}\hbar v_{\rm D}) \approx 2.2/\kappa_{\rm r}$.

The symmetric and antisymmetric pseudospin-pseudospin interactions can be decomposed as following:

$$\mathcal{J}_\ell(k-k') \equiv \mathcal{J}_{\ell,{\rm d}}(k-k') + \mathcal{J}_{\ell,{\rm x}}(k-k') \tag{38}$$

and

$$\mathcal{D}_2(k-k') \equiv \mathcal{D}_{2,{\rm d}}(k-k') + \mathcal{D}_{2,{\rm x}}(k-k')~. \tag{39}$$

The *direct* contributions are given by:

$$
\begin{aligned}
\mathcal{J}_{1,\mathrm{d}}(k-k') &= \frac{\mathcal{N}}{L^2}\sum_q e^{i\ell_B^2 q(k-k')} v_q |\mathcal{F}_{(+,M+1),(+,M)}(q\hat{\mathbf{y}})|^2 \ , &\text{(40a)}\\
\mathcal{J}_{2,\mathrm{d}}(k-k') &= 0 \ , &\text{(40b)}\\
\mathcal{J}_{3,\mathrm{d}}(k-k') &= \frac{\mathcal{N}}{4L^2}\sum_q e^{i\ell_B^2 q(k-k')} v_q \big[\mathcal{F}_{(+,M+1),(+,M+1)}(q\hat{\mathbf{y}}) \\
&- \mathcal{F}_{(+,M),(+,M)}(q\hat{\mathbf{y}})\big]^2 \ , &\text{(40c)}\\
\mathcal{D}_{2,\mathrm{x}}(k-k') &= \frac{\mathcal{N}}{2L^2}\sum_q e^{i\ell_B^2 q(k-k')} v_q \mathcal{F}_{(+,M+1),(+,M)}(q\hat{\mathbf{y}}) \\
&\times \ \big[\mathcal{F}_{(+,M+1),(+,M+1)}(q\hat{\mathbf{y}}) - \mathcal{F}_{(+,M),(+,M)}(q\hat{\mathbf{y}})\big] \ . &\text{(40d)}
\end{aligned}
$$

The *exchange* contributions are given by:

$$
\begin{aligned}
\mathcal{J}_{1,\mathrm{x}}(k-k') &= -\frac{\mathcal{N}_\phi}{2L^2}\sum_{\mathbf{q}} v_{\mathbf{q}}\big\{\mathcal{F}_{(+,M+1),(+,M+1)}(\mathbf{q})\mathcal{F}_{(+,M),(+,M)}(\mathbf{q}) \\
&- \ \mathrm{Re}^2\big[\mathcal{F}_{(+,M+1),(+,M)}(\mathbf{q})\big]\big\}\delta_{q_x,k-k'} \ , &\text{(41a)}\\
\mathcal{J}_{2,\mathrm{x}}(k-k') &= -\frac{\mathcal{N}_\phi}{2L^2}\sum_{\mathbf{q}} v_{\mathbf{q}}\big\{\mathcal{F}_{(+,M+1),(+,M+1)}(\mathbf{q})\mathcal{F}_{(+,M),(+,M)}(\mathbf{q}) \\
&+ \ \mathrm{Re}^2\big[\mathcal{F}_{(+,M+1),(+,M)}(\mathbf{q})\big]\big\}\delta_{q_x,k-k'} \ , &\text{(41b)}\\
\mathcal{J}_{3,\mathrm{x}}(k-k') &= \frac{\mathcal{N}_\phi}{4L^2}\sum_{\mathbf{q}} v_{\mathbf{q}}\big[2|\mathcal{F}_{(+,M+1),(+,M)}(\mathbf{q})|^2 - |\mathcal{F}_{(+,M+1),(+,M+1)}(\mathbf{q})|^2 \\
&- \ |\mathcal{F}_{(+,M),(+,M)}(\mathbf{q})|^2\big]\delta_{q_x,k-k'} \ , &\text{(41c)}\\
\mathcal{D}_{2,\mathrm{x}}(k-k') &= -\frac{\mathcal{N}_\phi}{2L^2}\sum_{\mathbf{q}} v_{\mathbf{q}}\big[\mathcal{F}_{(+,M+1),(+,M+1)}(\mathbf{q}) + \mathcal{F}_{(+,M),(+,M)}(\mathbf{q})\big] \\
&\times \ \mathrm{Re}\big[\mathcal{F}_{(+,M+1),(+,M)}(\mathbf{q})\big]\,\delta_{q_x,k-k'} \ , &\text{(41d)}
\end{aligned}
$$

where $\mathcal{N}_\phi$ has been introduced in the main text.

Supplementary Figures 2 (**a**), (**b**), (**c**) and (**d**) show the Fourier transforms

$$
\widetilde{\mathcal{J}}_\ell(q) \equiv \frac{1}{\mathcal{N}_\phi}\sum_k \mathcal{J}_\ell(k) e^{-iqk\ell_B^2} \qquad (42)
$$

and

$$
\widetilde{\mathcal{D}}_2(q) \equiv \frac{1}{\mathcal{N}_\phi}\sum_k \mathcal{D}_2(k) e^{-iqk\ell_B^2} \ , \qquad (43)
$$

for $M = 1$ (red solid line) and $M = 2$ (blue dashed line). In the $q \to 0$ limit we obtain: $\widetilde{\mathcal{D}}_2(0) = 0$, $\widetilde{\mathcal{J}}_\ell(0) = \widetilde{\mathcal{J}}_{\ell,\mathrm{x}}(0) \neq 0$ for any ℓ, and $\widetilde{\mathcal{J}}_1(0) = \widetilde{\mathcal{J}}_2(0)$. Moreover, we find that

$$a_{\mathrm{ee}} = 2\big[\widetilde{\mathcal{J}}_3(0) - \widetilde{\mathcal{J}}_1(0)\big] \ . \tag{44}$$

Supplementary Note 5

The correction Δ_{ee} in Eq. (35) to the cyclotron transition energy is related to the renormalization [6, 7, 8] of the Dirac velocity v_{D} due to exchange interactions, which occurs also in the absence of a magnetic field. It is well-known [9, 10, 11] that Δ_{ee} is logarithmically divergent, i.e.

$$\Delta_{\mathrm{ee}} = \alpha_{\mathrm{ee}} \frac{\Omega_M}{8} \left[\ln(n_{\mathrm{max}}) + C_M\right] \ , \tag{45}$$

where n_{max} is a cut-off defined below and the constant C_M depends on the highest-occupied LL with index M. For example, $C_0 \simeq -1.017$ and $C_1 \simeq -2.510$. The Dirac model applies over a large but finite energy region, so we define a high-energy cut-off W in valence band. At any given magnetic field B, the integer n_{max} represents the number of LLs in the valence band with energy larger than W, i.e.

$$n_{\mathrm{max}} = \frac{B_W}{B} \ , \tag{46}$$

where $B_W = eW^2/(2c\hbar v_{\mathrm{D}})$, such that $W = \sqrt{2}\hbar v_{\mathrm{D}}\sqrt{n_{\mathrm{max}}}/\ell_B$. We follow Shizuya [11] in writing the correction to cyclotron transition energy in terms of the renormalized Dirac velocity: $\Omega_M = \sqrt{2}\hbar v_{\mathrm{D}}^\star/\ell_B$ and $v_{\mathrm{D}}^\star = v_{\mathrm{D}} + \delta v_{\mathrm{D}}$. The quantity v_{D} is the bare Dirac velocity and $\delta v_{\mathrm{D}} = \alpha_{\mathrm{QED}} c/(8\kappa_{\mathrm{r}})[\ln(B_W/B) + C_M]$ is the correction to the Dirac velocity, where $\alpha_{\mathrm{QED}} = e^2/(\hbar c) \simeq 1/137$ is the QED fine-structure constant. This allows us to fix $B_W = 450$ Tesla to make sure that $v_{\mathrm{D}}^\star$ matches the value $v_{\mathrm{D}}^\star = 1.12 \times 10^6$ m/s measured [12] from the intraband $0 \to 1$ cyclotron transition energy at $B = 18$ Tesla and in a sample with $\kappa_{\mathrm{r}} = 5$. We have fixed the bare Dirac velocity to $v_{\mathrm{D}} = c/300$.

Supplementary Note 6

In this Supplementary Note we discuss the relative role of symmetric and antisymmetric pseudospin-pseudospin interactions in determining the precise form of the phase diagrams shown in Figure 2 of the main text.

We start by setting to zero all the symmetric interactions in the energy functional introduced in Eq. (7) of the main text: $\mathcal{J}_\ell = 0$. In this case, we find that the unstable regions (grey-shaded regions in Figure 2 of the main text) considerable expand. This is illustrated for the case $\kappa_{\rm r} = 15$ and $M = 1$ in Supplementary Figure 3.

Supplementary Note 7

In this Supplementary Note we explain more in detail the approach we have followed to find the elementary excitations of the polariton fluid.

In the spin-chain language introduced in the main text, the state $|\psi\rangle$ of the homogeneous fluid phase represents a *collinear ferromagnet* in which the expectation value of the spin operator $\mathbf{S}(\mathbf{q}) \equiv [S_1(\mathbf{q}), S_2(\mathbf{q}), S_3(\mathbf{q})]^{\rm T}$ is non-zero only at $\mathbf{q} = \mathbf{0}$ and oriented along the direction

$$\hat{\mathbf{z}}' = [-\cos(\phi)\sin(\theta), -\sin(\phi)\sin(\theta), \cos(\theta)]^{\rm T} \ , \tag{47}$$

i.e. $\langle\psi|\mathbf{S}(\mathbf{q})|\psi\rangle = -\hat{\mathbf{z}}'\sqrt{\mathcal{N}}\delta_{\mathbf{q},0}$. The collection of units vectors $\hat{\mathbf{x}}'$, $\hat{\mathbf{y}}'$, $\hat{\mathbf{z}}'$ with

$$\hat{\mathbf{x}}' = [\cos(\phi)\cos(\theta), \sin(\phi)\cos(\theta), \sin(\theta)]^{\rm T} \ , \tag{48}$$

and

$$\hat{\mathbf{y}}' = [-\sin(\phi), \cos(\phi), 0]^{\rm T} \ , \tag{49}$$

forms an orthonormal set, which will be used below to construct low-energy collective excitations above the ground state $|\psi\rangle$.

To study the collective mode spectrum, we assume that $|\psi\rangle$ is subject to an infinitesimal pertubation, which induces a small change $|\delta\psi\rangle$, i.e. we write $|\psi'\rangle = |\psi\rangle + |\delta\psi\rangle$. The most general infinitesimal change $|\delta\psi\rangle$ *orthogonal* to $|\psi\rangle$ can be written as a superposition of low-energy single-particle excitations [13]

$$|\delta\psi\rangle = \left(\sum_{\nu,\mathbf{q}} \alpha_{\mathbf{q}\nu} a^\dagger_{\mathbf{q},\nu} + \sum_{\xi,\mathbf{q}} r_{\mathbf{q}\xi} \bar{\rho}^{\xi\xi}_{+}(-\mathbf{q})\right)|\psi\rangle \ , \tag{50}$$

where $\bar{\rho}^{\xi\xi}_{+}(\mathbf{q}) = \bar{\rho}^{\xi\xi}(\mathbf{q}) \cdot (\hat{\mathbf{x}}' + i\hat{\mathbf{y}}')/2$ and $\bar{\rho}^{\xi\xi}(\mathbf{q}) \equiv [\bar{\rho}^{\xi\xi}_1(\mathbf{q}), \bar{\rho}^{\xi\xi}_2(\mathbf{q}), \bar{\rho}^{\xi\xi}_3(\mathbf{q})]^{\rm T}$, with $\bar{\rho}^{\xi\xi}_m(\mathbf{q})$ as in Eq. (17). In writing Eq. (50) we have assumed that the infinitesimal pertubation does not induce any spin-flip or intervalley scattering process.

In the time-dependent Hartree-Fock (or generalized random phase) approximation [13], the spin wave spectrum can be calculated by making use of the Heisenberg equation of motion (EOM) for the expectation value of an operator $\mathcal{O}$, evaluated on the state $|\psi'\rangle$, i.e. $i\hbar\partial_t\langle\psi'|\mathcal{O}|\psi'\rangle = \langle\psi'|[\mathcal{O},\mathcal{H}]|\psi'\rangle$, where the total Hamiltonian $\mathcal{H}$ is reported in Eq. (1) of the main text. Writing EOMs for $\mathcal{O} = a_{\mathbf{q},\nu}$, $\bar{\rho}_{-}^{\xi\xi}(\mathbf{q})$, $a^{\dagger}_{\mathbf{q},\nu}$, and $\bar{\rho}_{+}^{\xi\xi}(\mathbf{q})$ and keeping terms up to linear order in $\alpha_{\mathbf{q}\nu} = \langle a_{\mathbf{q},\nu}\rangle$ and $r_{\mathbf{q}\xi} = \langle\bar{\rho}_{-}^{\xi\xi}(\mathbf{q})\rangle$, we find a homogeneous system of first-order linear differential equations. Eigenmodes are found by replacing $i\hbar\partial_t \to \Omega$. We obtain an eigenvalue problem, whose solution gives the spin wave spectrum. We find six independent collective modes, four due to the fourfold spin-valley degeneracy and the remaining two due to the two possible light polarizations. Only two collective modes are *hybrid* in that they contain both light and matter components. They are composed by a left-handed photon $a_{\mathbf{q},\mathrm{L}}$ and a bright collective matter mode $S'_{-}(\mathbf{q}) = \mathbf{S}(\mathbf{q})\cdot(\hat{\mathbf{x}}' - i\hat{\mathbf{y}}')/2$. In this subspace of hybrid modes, the eigenvalue problem reduces to $(\Omega_{\mathbf{q}}\mathbb{1} - \mathbf{M})\mathbf{v} = 0$ where $\mathbb{1}$ is the 4×4 identity and the 4×4 matrix $\mathbf{M}$ is reported in Eq. (22) of the main text.

Supplementary References

[1] Castro Neto, A. H., Peres, N. M. R., Novoselov, K. S. & Geim, A. K. The electronic properties of graphene. *Rev. Mod. Phys.* **81**, 109-162 (2009).

[2] M.I. Katsnelson, *Graphene: Carbon in Two Dimensions* (Cambridge University Press, Cambridge, 2012).

[3] Giuliani, G. F. & Vignale, G. *Quantum Theory of the Electron Liquid* (Cambridge University Press, Cambridge, 2005).

[4] Goerbig, M. O. Electronic properties of graphene in a strong magnetic field. *Rev. Mod. Phys.* **83**, 1193-1243 (2011).

[5] I. S. Gradshteyn, and I. M. Ryzhik, *Table of Integrals, Series and Products* (Academic Press, San Diego, 2000).

[6] González, J., Guinea, F. & Vozmediano, M. A. H. Marginal-Fermi-liquid behavior from two-dimensional Coulomb interaction. *Phys. Rev. B* **59**, R2474 (1999).

[7] Borghi, G., Polini, M., Asgari, R. & MacDonald, A. H. Fermi velocity enhancement in monolayer and bilayer graphene. *Solid State Commun.* **149**, 1117-1122 (2009).

[8] Elias, D. C., Gorbachev, R. V., Mayorov, A. S., Morozov, S. V., Zhukov, A. A., Blake, P., Ponomarenko, L. A., Grigorieva, I. V., Novoselov, K. S., Guinea, F. & Geim, A. K. Dirac cones reshaped by interaction effects in suspended graphene. *Nature Phys.* **7**, 701-704 (2011).

[9] Iyengar, A., Wang, J., Fertig, H. A. & Brey, L. Excitations from filled Landau levels in graphene. *Phys. Rev. B* **75**, 125430 (2007).

[10] Bychkov, Yu. & Martinez, G. Magnetoplasmon excitations in graphene for filling factors $\nu \leq 6$. *Phys. Rev. B* **77**, 125417 (2008).

[11] Shizuya, K. Many-body corrections to cyclotron resonance in monolayer and bilayer graphene. *Phys. Rev. B* **81**, 075407 (2010).

[12] Jiang, Z., Henriksen, E. A., Tung, L. C., Wang, Y.-J.,Schwartz, M. E., Han, M. Y., Kim, P. & Stormer, H. L. Infrared spectroscopy of Landau levels of graphene. *Phys. Rev. Lett.* **98**, 197403 (2007).

[13] Negele, J. W. & Orland, H. *Quantum Many-Particle Systems* (Westview Press, Boulder, 1988).
